# Mortality From Acute Poisoning in Urmia: A Three- Year Retrospective Study

**DOI:** 10.5812/ircmj.1887

**Published:** 2012-12-06

**Authors:** Behzad Boshehri, Saleh Salimi, Sara Ranjbar

**Affiliations:** 1 Department of Toxicology and Forensic Medicine, Urmia University of Medical Sciences, Urmia, IR Iran; 2 Nursing and Midwifery School, IAU of Urmia Branch, Urmia, IR Iran; 3 Urmia University of Medical Sciences, Urmia, IR Iran

**Keywords:** Mortality, Poisoning

Dear Editor

Poisoning is the worldwide problem and its mortality has increased dramatically all around the world. Whereas unintentional poisoning deaths were most often a consequence of illicit drugs in prior decades, since the latter half of the 1990s, medications began to account for a greater number of fatal poisonings (1). In Iran, like most other developing countries, it is mostly attributed to deliberate self-poisoning (2). Poisoning from pesticides is the most common cause of death in some developing countries, while in many industrialized countries and metropolitan areas accidental opiates overdose mortality is the most common cause (3). Early correct diagnosis and appropriate treatment of poisoned cases are often live saving. In this retrospective study, all of the patients with the diagnosis of acute poisoning presenting to the ED of the Talegani Hospital in Urmia, a reference hospital for poisoning at west Azerbaijan province of Iran, were evaluated from March 2007 to March 2009. Records of all patients with acute poisoning diagnosis who were admitted to ED and then transferred to poisoning unit or MICU were reviewed. Data regarding the age, gender, marital status, occupation, socio-economic status, involved toxic agents, route of intake, duration of treatment, admission status, final outcome, and cause of death (if any) collected from the medical records. Patients with idiosyncratic or adverse reaction to prescribed drugs excluded from the study. The research was approved by Human Subjects committee of Medicine College of Medical Sciences University of Urmia. From total of 4062 poisoned cases in three years, 70 persons (1.72%) were expired. Of those who died, the majority were young adults (38.6%), 65.71% were males, and 61.43% were married. The ratio of male to female was 1.92:1. Most of died cases were poisoned intentionally (62.86%), and the most involved toxic agents were: agrochemicals (40%), opium (28.58%), and drugs (17.15%). Among those who died due to drug poisoning, most of cases had been consumed a mix of a few drugs (33/83%). The most frequently involved medicinal drugs were benzodiazepines (diazepam and chlordiazepoxide), TCA (mainly nortriptyline), Tramadol, cardiovascular medications (losartan and amlodipine), and metformin. Mortality from opiates and drugs were common in males than females. Most of the deaths took place in summer (42.86%) and spring (37.14%), and in the first 24 hours after admission (41.43%). Delays in presentation were common, only 40% of the patients arrived at hospital on or before 3 hours. The route of administration was as follows: 71.14% orally, 21.43% by intravenous injection, and 1.43% by inhalation. The most frequent complication was respiratory arrest (47.14%) followed by cerebral disorders (24.30%). The leading occupations were: unemployed house-wives (27.15%), self- employed (20%), male farmers (14.29%), jobless unemployed (11.42%), and students (7.15%).Findings showed that mortality from acute poisoning in Urmia is similar to those of the developed countries in which it were reported less than 2% and is less than the rates of the developing countries in which it were reported between 2 to 15% (2-5). The majority of deceased cases were young adults which were in agreement with the findings of the studies in China, Ireland, and South Africa (5-7). In accordance with the findings of the prior studies mortality among men was greater than women (6). The most common cause of deaths among men was opiates (25.71%). In view of the fact that, in Iran, there is more than three millions addict that most of them are male, these findings can be justified. Among women, the most common cause of death was agrochemicals, mostly organophosphates (21.42%). This finding is in consistent with the prior researches in developing countries and is justifiable since Urmia, and the West Azerbayjan, in general, is an agricultural place (8). In the present study, drug–induced mortality were in third rank while in some studies conducted in western communities it was reported in first rank (9). Surprisingly, the mortality rate among married was higher than singles. The results illustrated that the majority of deceased cases consumed poison intentionally. This finding is in agreement with the findings of previous studies in Iran but there is remarkable dissimilarity with the findings of some studies carried out in the U.S. and Europe in which poisoning, in most of the age groups, is unintentionally (4, 9, 10). Among occupations, unemployed housewives and farmers have an increased risk for deliberate self-poisoning and the death resulting from it (27.15% and 14.29%). This finding is comparable with the findings of a study in china, but it might be a reflection of improper circumstances of the unemployed house-wives in Iran (7). Although mortality rate of acute poisoning is comparable with those of the developed countries, precise and on time interventions may reduce complications and death rate more. The other priority in this region should be the prevention of poisoning due to opiates and agrochemicals.
